# γ-Tocotrienol Sensitises Colorectal Cancer to PD-1 Blockade by Enhancing MHC-I-Associated Tumour Immune Visibility and CD8^+^ T Cell-Related Antitumour Immunity

**DOI:** 10.3390/biom16070964

**Published:** 2026-06-30

**Authors:** Haixia Wang, Xiaohe Chu, Can Xu, Zilong Li, Ling Xie

**Affiliations:** 1 Collaborative Innovation Center of Yangtze River Delta Region Green Pharmaceuticals, Zhejiang University of Technology, Hangzhou 310014, China; 2 Hangzhou Institute of Medicine (HIM), Chinese Academy of Sciences, Hangzhou 310022, China

**Keywords:** γ-tocotrienol, natural-source antitumour agent, colorectal cancer, cancer immunotherapy

## Abstract

γ-Tocotrienol (γ-T3), a naturally occurring vitamin E isoform from plant-derived sources, has attracted attention as an antitumour agent. However, whether γ-T3 can enhance antitumour immunity and improve immune checkpoint blockade remains unclear. Here, using colorectal cancer (CRC) models, we found that γ-T3 suppressed tumour growth in immunocompetent MC38 and CT26 mouse models, whereas this effect was markedly weakened in immunodeficient hosts, indicating that its in vivo antitumour activity is closely associated with host immunity. Combination treatment with γ-T3 and programmed cell death protein 1 (PD-1) blockade further improved tumour control, accompanied by enhanced CD8^+^ T cell effector function, reduced regulatory T cell abundance, and tumour-associated macrophage remodelling towards an antitumour phenotype. Immune cell depletion experiments confirmed that CD8^+^ T cells are the principal effector cells mediating γ-T3-associated tumour suppression. Mechanistically, HSPA4 was identified as a candidate γ-T3-associated protein potentially linked to MHC-I-related immune-recognition features. γ-T3 promoted the expression of Psmb8 and Tap2 and increased MHC-I surface levels on tumour cells, accompanied by increased sensitivity of tumour cells to activated CD8^+^ T cell-mediated growth inhibition. These findings support γ-T3 as a naturally derived immune-sensitising agent for improving PD-1 blockade therapy in CRC.

## 1. Introduction

CRC ranks among the most frequent cancers globally and is a major contributor to cancer mortality [1]. Although chemotherapy, targeted therapy, and immune checkpoint blockade have continuously expanded the therapeutic options for colorectal cancer, the long-term benefits for patients remain limited by treatment resistance, recurrence and metastasis, heterogeneity of therapeutic response, and treatment-related toxicities [2]. Therefore, the discovery of antitumour-active molecules from natural sources with well-defined mechanisms of action, favourable safety profiles, and a capacity to boost current therapies continues to be a key research goal for preventing and treating colorectal cancer [3,4,5]. Naturally derived small molecules may not only directly inhibit tumour cell proliferation or induce cell death but also potentiate the response to current therapeutic strategies by modulating the tumour immune microenvironment and enhancing antitumour immune responses [4,6,7].

γ-T3, the γ isoform of the tocotrienol subclass within the vitamin E family, is naturally present in plant-derived sources, such as palm oil, rice bran oil, annatto seeds, and certain cereals [8], and exhibits diverse biological activities, including antitumour, anti-inflammatory, antioxidant, and immunomodulatory effects [9]. Previous studies have mainly focused on the regulation of tumour cell-intrinsic processes by γ-T3, including tumour cell proliferation, programmed cell death, and inflammation-related signalling pathways, and they have suggested its potential antitumour activity in colorectal cancer [10]. Although a limited number of studies have preliminarily reported that γ-T3 or tocotrienol-related formulations may affect T cell/Treg-associated immune status or enhance dendritic cell vaccine-induced tumour-specific cytotoxic responses, the available immune-related evidence remains largely confined to preliminary immunophenotypic observations and has mainly been generated in the contexts of breast cancer models, dietary supplementation, or vaccine adjuvant strategies [11,12]. Therefore, whether single-component γ-T3 can enhance the response of colorectal cancer to PD-1 blockade, and whether this effect is mediated through CD8^+^ T cell-dependent antitumour immunity and MHC-I-associated tumour immune visibility, remains to be further elucidated.

Immune checkpoint blockade provides an important therapeutic strategy for colorectal cancer by restoring the antitumour activity of T cells [13,14]. However, the response of patients with colorectal cancer to PD-1/PD-L1 blockade demonstrates marked heterogeneity. While patients with dMMR/MSI-H (deficient mismatch repair/microsatellite instability-high) tumours are generally more likely to derive benefit, primary low response or acquired resistance may still occur; conversely, the majority of patients with pMMR/MSS (proficient mismatch repair/microsatellite stable) tumours exhibit a limited overall response [3,15,16]. These observations suggest that the suboptimal efficacy of immunotherapy in colorectal cancer is not only owing to insufficient restoration of T cell response but also closely linked to the ability of tumour cells to continuously present antigenic signals recognisable by the immune system. Therefore, enhancing the immunological visibility of tumour cells may represent an important strategy to render colorectal cancer more amenable to PD-1 blockade-based treatment.

Immune visibility of cancer cells hinges on MHC-I-dependent antigen display [17]. CD8^+^ T cells execute their cytotoxic functions by recognising peptide–MHC-I complexes on the tumour cell surface; thus, MHC-I antigen presentation represents a critical link connecting intrinsic tumour cell abnormalities with adaptive immune recognition [14]. Tumour cells can evade immune surveillance by reducing the expression of antigen processing-related molecules, impairing peptide loading, interfering with MHC-I trafficking, or promoting its degradation [7,14,18,19]. Accordingly, enhancing antigen processing, peptide transport, and surface presentation of MHC-I is expected to increase the vulnerability of tumour cells to CD8^+^ T cell-mediated growth inhibition and provide a more robust upstream antigen recognition input for PD-1/PD-L1 blockade therapy.

MHC-I antigen presentation depends on the surface display of MHC class I molecules themselves, coupled with the intracellular supply of antigenic peptides [17,20]. The antigenic peptides presented by MHC-I are primarily derived from the degradation products of intracellular proteins, including misfolded proteins, short-lived proteins, defective ribosomal products, and other protein substrates that enter the ubiquitin–proteasome system [20,21,22,23]. Consequently, protein quality control, proteasomal processing, and peptide transport collectively affect the efficiency with which antigenic peptides enter the MHC-I presentation pathway [24]. A previous study has shown that modulating chaperone-mediated protein homeostasis can reshape the MHC-I antigenic peptide presentation landscape in tumour cells, suggesting that the protein homeostasis network may be involved in the regulation of tumour antigen presentation and immunogenicity [25]. HSPA4, also known as APG-2, belongs to the Hsp110 family and serves as a critical nucleotide exchange factor associated with the Hsp70 chaperone network. It participates in protein conformation maintenance, misfolded protein processing, inhibition of protein aggregation, and the regulation of protein homeostasis under cellular stress conditions [26]. Therefore, HSPA4 may be positioned at the regulatory interface between protein quality control and antigenic peptide supply, and it may indirectly be involved within the control of MHC-I antigenic peptide source by influencing the efficiency with which aberrant protein substrates enter the ubiquitin–proteasome system. Nevertheless, whether HSPA4 is involved in the modulation of antigen processing and MHC-I display in colorectal cancer cells, and whether this process can be modulated by γ-T3, remains to be systematically investigated.

Based on the aforementioned issues, we investigated whether γ-T3 can enhance MHC-I-associated tumour immune visibility and improve CD8^+^ T cell-related antitumour immunity in colorectal cancer. We systematically evaluated the immune-dependent antitumour effects of γ-T3 and its combinational efficacy with PD-1 blockade therapy. Furthermore, we explored HSPA4 as a candidate γ-T3-associated protein potentially linked to protein homeostasis and MHC-I-related immune-recognition features. Via assessment of the tumour immune microenvironment, immune cell depletion experiments and in vitro T cell co-culture assays, we delineated the role of CD8^+^ T cells in this process. Furthermore, focusing on HSPA4 and its associated regulation of protein homeostasis, this study explored the potential mechanisms by which γ-T3 affects antigen processing, peptide transport, and surface presentation of MHC-I. Together, these analyses provide experimental support for the potential use of γ-T3 as a naturally derived immune-sensitising molecule in combination with PD-1 blockade.

## 2. Materials and Methods

### 2.1. Reagents and Antibodies

γ-T3 was acquired via MedChemExpress (Monmouth Junction, NJ, USA); 5-fluorouracil (5-FU) was obtained from Sigma-Aldrich (St. Louis, MO, USA). Foetal bovine serum, RPMI-1640 medium, penicillin-streptomycin solution, and trypsin-EDTA were purchased from Gibco (Grand Island, NY, USA). Phosphate-buffered saline (PBS) was obtained from HyClone (Logan, UT, USA). Anti-mouse PD-1 antibody, anti-mouse CD4 depletion antibody, and anti-mouse CD8α depletion antibody were purchased from Bio X Cell Bio X Cell (Lebanon, NH, USA). PLX3397 was obtained from Selleck Chemicals (Houston, TX, USA). Antibodies used for flow cytometry included Zombie BV510 viability dye, as well as antibodies against CD45, CD3, CD4, CD8, CD25, FOXP3, IFN-γ, Granzyme B, CD11b, F4/80, CD86, CD206, CD11c, CD80, and MHC-II, which were purchased from BioLegend (San Diego, CA, USA), BD Biosciences (Franklin Lakes, NJ, USA), or Invitrogen (Carlsbad, CA, USA). TRIzol reagent was purchased from Thermo Fisher Scientific (Waltham, MA, USA); Vazyme (Nanjing, China) supplied the reverse transcription kit and the SYBR Green qPCR master mix. Servicebio (Waltham, MA, USA) provided the qPCR primer synthesis. The mouse IFN-γ ELISA kit was purchased from Thermo Fisher Scientific.

### 2.2. Cell Lines and Cell Culture

MC38 and CT26, two mouse colorectal cancer cell lines, were obtained from the Shanghai Cell Bank of the Chinese Academy of Sciences. The cells were maintained in RPMI-1640 complete medium supplemented with 10% foetal bovine serum and 1% penicillin-streptomycin solution, and they were routinely passaged in a humidified incubator set at 37 °C with 5% CO_2_. The cells inoculated into animals were all selected from those in the logarithmic growth phase, with uniform morphology, good adhesion state, and no contamination. After digestion and collection, the cells were washed 2–3 times with sterile PBS to remove the residues of culture medium and pancreatic enzymes, and trypan blue was used to determine cell viability. Cell suspensions with good viability and no obvious masses were selected for subcutaneous inoculation in mice.

### 2.3. Experimental Animals

Female C57BL/6, BALB/c, and NOD-SCID mice (6–8 weeks of age) came from Shanghai SLAC Laboratory Animal Co., Ltd. (Shanghai, China). They were kept under SPF conditions at the Hangzhou Institute of Medicine, Chinese Academy of Sciences (25 °C, ~50% humidity), with free access to food and water. All animal studies followed applicable ethical standards for laboratory animal care and were approved by the institute’s Ethics Committee.

### 2.4. Establishment of an Immunocompetent Syngeneic Subcutaneous Transplantation Tumour Model and Evaluation of γ-T3 Monotherapy

To evaluate the in vivo antitumour activity of γ-T3 in immunocompetent hosts, BALB/c-CT26 and C57BL/6-MC38 syngeneic subcutaneous transplantation tumour models were established. CT26 cells (3 × 10^5^ cells/100 μL) and MC38 cells (1 × 10^6^ cells/100 μL) were inoculated into the right flank of BALB/c and C57BL/6 mice, respectively. After tumour size attained about 80–100 mm^3^, these animals were randomly separated into four groups according to their tumour volume: control group, γ-T3 20 mg/kg group, γ-T3 40 mg/kg group, and 5-FU 25 mg/kg group. Both γ-T3 and 5-FU were injected intraperitoneally every other day. Control mice were given the same amount of corn oil. During the treatment period, the longest diameter, shortest diameter, and the body mass of these animals were recorded every 2nd day, and the general condition of the mice was observed. Tumour volume was calculated as V = (L × W^2^)/2, where L represents the longest diameter and W represents the shortest diameter. At study completion, the animals were sacrificed, and tumour samples were collected for further assays.

### 2.5. Establishment of Subcutaneous Tumour Models in NOD-SCID Immunodeficient Hosts

To assess whether the antitumour effect of γ-T3 depends on the host immune system, NOD-SCID-MC38 and NOD-SCID-CT26 subcutaneous tumour models were established. Cultured MC38 or CT26 cells collected during the exponential growth stage were centrifuged and re-suspended in sterilised phosphate-buffered saline (PBS), then injected beneath the skin into the right dorsal region of NOD-SCID mice. Once the resulting tumours had grown to a size of nearly 80–100 mm^3^, the animals were assigned to two cohorts based on tumour dimensions: a control group and a group receiving 20 mg/kg of γ-T3. γ-T3 was administered by intraperitoneal injection once every 2 days, while the control group received an equal volume of corn oil. Tumour volume was measured regularly during treatment, and the physical state of the mice was evaluated. Upon study termination, all animals were humanely euthanised, after which neoplastic specimens were resected to enable pharmacodynamic assessment.

### 2.6. Subcutaneous Tumour Models for γ-T3 Plus PD-1 Checkpoint Inhibition

For evaluating the combinatorial anticancer effectiveness of γ-T3 plus PD-1 inhibition, combination treatment experiments were performed in immunocompetent C57BL/6-MC38 and BALB/c-CT26 subcutaneous tumour models. Tumour inoculation was performed as described above. After tumour dimensions reached roughly 80–100 mm^3^, tumour-bearing mice were divided at random into the control group, γ-T3 monotherapy group, anti-PD-1 antibody group, or combination treatment group. Mice in the γ-T3 monotherapy group received γ-T3 at 20 mg/kg by intraperitoneal injection, whereas those in the anti-PD-1 antibody group received anti-mouse PD-1 antibody at 5 mg/kg by intraperitoneal injection. Mice in the combination treatment group received both γ-T3 and anti-mouse PD-1 antibody by intraperitoneal injection. All treatments were administered once every 2 days, and control mice received an equal amount of corn oil by intraperitoneal injection. During treatment, tumour size and body weight were assessed every 48 h, and the overall well-being of the animals was monitored daily to evaluate treatment tolerability and potential toxicity.

### 2.7. Preparation of Single-Cell Suspensions Derived from Tumour Tissues and Lymph Nodes, Followed by Flow Cytometric Analysis of Immune Cells

To analyse the composition and functional phenotypes of immune cells in tumour tissues and tumour-draining lymph nodes, mice were euthanised at the experimental endpoint, and tumour tissues and corresponding tumour-draining lymph nodes were collected under sterile conditions. Tumour tissues were minced in pre-cooled RPMI-1640 medium and digested in RPMI-1640 digestion buffer containing 2% FBS and collagenase D at 37 °C for 25 min. The digested tissue suspension was passed through a 70 μm cell strainer and centrifuged at 850× *g* for 10 min at 4 °C. Red blood cell lysis was performed when necessary, after which cells were resuspended in flow cytometry staining buffer and counted. Single-cell suspensions were acquired by mechanically disrupting tumour-draining lymph nodes using a 70 μm strainer, followed by centrifugation, resuspension, and cell counting.

All samples were first subjected to live/dead staining and Fc receptor blocking. Cells were exposed to Zombie BV510 viability dye for 10–15 min under ambient conditions without light, rinsed, and then treated with anti-mouse CD16/32 antibody for 10–15 min at 4 °C. The corresponding antibody mixtures were then added according to the different staining panels, then kept for 25–30 min at 4 °C without light.

For T cell functional analysis, tumour-derived single-cell suspensions were stimulated with Cell Activation Cocktail containing Brefeldin A for 4 h at 37 °C in 5% CO_2_, followed by surface and intracellular staining. CD4^+^ T cells and CD8^+^ T cells were defined as CD45^+^CD3^+^CD4^+^ and CD45^+^CD3^+^CD8^+^ cells, respectively, and IFN-γ and Granzyme B expression was analysed within these populations. Regulatory T cells were defined as CD45^+^CD3^+^CD4^+^CD25^+^FOXP3^+^ cells. Tumour-associated macrophages were defined as CD45^+^CD11b^+^F4/80^+^ cells, and M1-like and M2-like polarisation phenotypes were assessed based on CD86 and CD206 expression, respectively. Dendritic cells were analysed using single-cell suspensions derived from tumour-draining lymph nodes and were gated as live CD45^+^CD11c^+^ cells. Dendritic cell maturation was assessed by the mean fluorescence intensity (MFI) of CD80, CD86, and MHC-II within the gated DC population. FlowJo software v10.9.0 was employed to analyse the flow cytometry data. Cellular debris was gated out according to FSC/SSC characteristics, and doublets were removed, and dead cells were excluded according to live/dead staining. T cells, regulatory T cells, tumour-associated macrophages, and dendritic cell maturation were then analysed within the live CD45^+^ cell population according to the corresponding gating strategies for each panel. The relevant gating strategies are shown in Figure S1A–D. The fluorescent labels, clone numbers, manufacturers and working concentrations of all antibodies are listed in Table S1.

### 2.8. Immune Cell Depletion

To identify the immune cell types essential for γ-T3-mediated tumour growth control in vivo, immune cell depletion experiments were conducted in the C57BL/6-MC38 syngeneic subcutaneous transplantation tumour model. The right flank of 6- to 8-week-old female C57BL/6 mice received a subcutaneous injection of the MC38 cells (1 × 10^6^ cells). Once the tumour size attained roughly 80–100 mm^3^, the mice were randomly assigned to the following groups: vehicle, γ-T3 alone, anti-CD4 antibody, anti-CD8 antibody, PLX3397 alone, and γ-T3 combined with anti-CD4 antibody, anti-CD8 antibody, or PLX3397, respectively.

For in vivo depletion, CD4^+^ T cells and CD8^+^ T cells were depleted using anti-CD4 or anti-CD8 antibodies, respectively, at a dose of 150 μg per mouse administered via intraperitoneal injection. Macrophages were inhibited by oral gavage of PLX3397 50 mg/kg. Immune cell depletion or inhibition was initiated 2 days before the first γ-T3 administration and subsequently maintained twice per week. Tumour sizes were measured every other day throughout treatment. To verify the efficiency of depletion or inhibition, spleens were collected from mice in an independent preliminary experiment, and single-cell suspensions were prepared. The proportions of CD45^+^CD3^+^CD4^+^ T cells, CD45^+^CD3^+^CD8^+^ T cells, and CD45^+^CD11b^+^F4/80^+^ macrophages were assessed by flow cytometry. By comparing the antitumour efficacy of γ-T3 under different immune cell depletion or inhibition conditions, the key immune cell types responsible for its in vivo pharmacological effect were evaluated.

### 2.9. Single-Cell RNA Sequencing Library Construction and Sequencing

Six- to eight-week-old female C57BL/6 mice were used to establish MC38 subcutaneous tumour models and were randomly assigned to the Control, γ-T3, PD-1 blockade, and γ-T3 + PD-1 blockade groups. At the end of treatment, tumour tissues from six mice in each group were pooled to prepare one single-cell suspension per group for exploratory single-cell RNA sequencing. Fresh tumour tissues were mechanically minced and enzymatically digested, followed by removal of tissue debris and cell aggregates. Cell viability assessment was performed before loading onto the Singleron Matrix single-cell processing system. Library construction and sequencing were conducted according to the manufacturer’s instructions. Detailed sample pooling and quality-control metrics are provided in Table S2.

Raw sequencing data were processed for quality control, alignment, UMI counting, and gene-expression matrix generation. Low-quality cells and lowly expressed genes were filtered out before downstream analysis. Downstream analyses were performed using Seurat v5.0.1 in R v4.3.2, including normalisation, highly variable gene selection, principal component analysis, dimensionality reduction, and clustering. Batch effects among samples were corrected using Harmony through the RunHarmony function. Cell types were annotated according to canonical marker genes and known lineage features.

Differentially expressed genes were identified within corresponding cell populations or subclusters using a threshold of |log_2_FC| > 0.26 and FDR < 0.05 [27]. Multiple-testing correction was performed using the Benjamini–Hochberg method. KEGG pathway-enrichment analysis was subsequently performed based on the identified differentially expressed genes. 

### 2.10. CD8^+^ T Cell Isolation, Activation, and Co-Culture

CD8^+^ T cells were isolated from mouse spleens by magnetic bead-based separation. Briefly, spleens were aseptically collected and mechanically dissociated through a cell strainer to obtain single-cell suspensions. Red blood cells were removed using red blood cell lysis buffer, and splenocytes were washed with PBS. CD8^+^ T cells were then isolated using a mouse CD8^+^ T cell isolation kit according to the manufacturer’s instructions. Purified CD8^+^ T cells were resuspended in RPMI-1640 complete medium supplemented with 10% foetal bovine serum and activated using ActBeads™ Mouse CD3/CD28 Activator beads for 24 h. For activation, CD3/CD28 activator beads were added at a bead-to-cell ratio of 1:1, and IL-2 was added to a final concentration of 100 U/mL. Cells were cultured at 37 °C in a humidified incubator containing 5% CO_2_. After activation, CD8^+^ T cells were collected, counted, and resuspended at the required concentration for subsequent co-culture experiments.

MC38 cells were seeded in parallel in 6-well and 96-well plates and cultured until they reached approximately 60–70% confluence. The cells were then pretreated with γ-T3 or vehicle control for 72 h. After γ-T3 pretreatment, MC38 cells were gently washed twice with sterile PBS to remove residual γ-T3 and detached cells. Fresh complete medium was then added before co-culture with activated CD8^+^ T cells. Activated CD8^+^ T cells were added to MC38 cells at an effector-to-target ratio of 1:1 and co-cultured for 24 h. The experimental groups were as follows: MC38 cells alone, γ-T3-pretreated MC38 cells, MC38 cells co-cultured with activated CD8^+^ T cells, and γ-T3-pretreated MC38 cells co-cultured with activated CD8^+^ T cells.

After 24 h of co-culture, culture supernatants from 6-well plates were collected for IFN-γ measurement. For the 96-well plates, non-adherent CD8^+^ T cells were carefully removed, and the remaining adherent MC38 cells were gently washed before assessment of relative tumour cell viability. The CD8^+^ T cells used in this assay were polyclonally activated by CD3/CD28 stimulation and were not generated against a defined tumour antigen. Therefore, this co-culture assay was used to evaluate activated CD8^+^ T cell-mediated tumour cell growth inhibition and IFN-γ release rather than antigen-specific cytotoxicity.

### 2.11. Detection of IFN-γ in Co-Culture Supernatants

To evaluate the activation level of CD8^+^ T cells in the co-culture system, culture supernatants were collected 24 h after co-culture and centrifuged at 1000× *g* for 10 min at 4 °C to remove residual cells and debris. The clarified supernatants were either assayed immediately or aliquoted and stored at −80 °C, avoiding repeated freeze–thaw cycles. The concentration of IFN-γ in the supernatants was determined using a mouse IFN-γ ELISA kit. Standard samples, blank controls, and test samples were added according to the manufacturer’s instructions, and the procedures of incubation, plate washing, colour development, and stop reaction were performed accordingly [28]. The absorbance was measured at 450 nm. The IFN-γ concentration values for individual samples were derived from a standard curve. Samples with values exceeding the linear range were re-assayed after appropriate dilution, and the actual concentration was calculated by multiplying with the dilution factor.

### 2.12. Cell Viability Assay

To assess the relative viability of adherent MC38 cells after co-culture, the remaining culture medium was removed, and the cells were gently washed with PBS to eliminate suspended CD8^+^ T cells. Afterwards, new medium with 10% CCK-8 was added, and then the mixture was incubated for 60 min at 37 °C protected from light. Absorbance was measured at 450 nm. After subtracting the background absorbance of blank wells, relative cell viability was normalised to the MC38 alone group using the following formula:

relative cell viability (%) = [(OD_experimental − OD_blank)/(OD_MC38 alone − OD_blank)] × 100%. All experiments were performed in triplicate.

### 2.13. Detection of Surface MHC-I Expression on Tumour Cells

MC38 and CT26 cells were exposed to graded γ-T3 doses. Following treatment, cells were collected and stained with a fluorescence-labelled anti-mouse MHC-I antibody. We examined the specimens via flow cytometry, and MHC class I abundance was reported either as mean fluorescence intensity or as normalised fluorescence values.

### 2.14. Limited Proteolysis–Mass Spectrometry Analysis

Limited proteolysis–mass spectrometry (LiP-MS) was applied to screen for potential γ-T3-binding proteins [29,30]. Following protein quantification of cell lysates, equal masses of total protein from each group were exposed to either γ-T3 or DMSO. Subsequently, proteinase K was added to perform limited proteolysis, and the reaction was terminated by heating. After complete trypsinization, the samples were acidified and desalted. Subsequent LC-MS/MS analysis was carried out using an Easy-nLC 1200 system interfaced with an Orbitrap Q Exactive Plus mass spectrometer. Differential LiP peptides were identified through label-free quantification and intergroup comparison and then mapped to their corresponding protein sequences to identify candidate γ-T3 target proteins and potential conformationally responsive regions.

### 2.15. Molecular Docking Analysis

The structural model of HSPA4 was obtained from the AlphaFold database, and the small-molecule structure of γ-T3 was downloaded from the PubChem database. After format conversion and structural preprocessing of the protein and ligand, AutoDock v1.2.5 Vina was employed to conduct molecular docking analysis. The docking results were visualised using PyMOL v2.5.4 and PLIP v2.3.0, and potential hydrogen bonds, hydrophobic interactions, and other non-covalent interactions between γ-T3 and HSPA4 were analysed.

### 2.16. Surface Plasmon Resonance Analysis

The direct binding between γ-T3 and HSPA4 was examined using a Biacore SPR system [31,32]. HSPA4 protein was immobilised onto the surface of a CM5 sensor chip via amine coupling, and various concentrations of γ-T3 were sequentially flowed over the flow channel and the reference channel. The association and dissociation processes were recorded in real time. Following reference subtraction and blank correction, the equilibrium dissociation constant (K_D) was calculated using Biacore Evaluation software v3.0.12. Both wild-type HSPA4 and the S323A mutant protein were analysed using the same procedure.

### 2.17. Total RNA Purification and Subsequent SYBR Green-Based Real-Time PCR

Cellular and tumoural RNA was isolated with TRIzol reagent. To prepare cDNA, we adhered to the reverse transcription kit’s manual, followed by SYBR Green-based quantitative real-time PCR, with β-actin acting as an internal housekeeping gene. Transcript levels were then quantified based on the 2^−ΔΔCt^ algorithm. The genes examined included H2-K1, H2-D1, Tap2, Psmb8, Hspa1a, and Hspb1. Primer sequences are listed in the Table S3.

### 2.18. Statistical Analysis

Statistical analyses were performed using GraphPad Prism 9.0 and R 4.1.3 software. Data are presented as mean ± standard deviation (SD). Comparisons between two groups were conducted using two-tailed unpaired Student’s *t*-tests. Comparisons among multiple groups were performed using one-way or two-way analysis of variance (ANOVA), followed by multiple comparisons correction as appropriate. A *p* value of <0.05 was considered statistically significant.

## 3. Results

### 3.1. γ-T3 Suppresses Colorectal Cancer Growth in an Immune-Dependent Manner

**Figure 1:**
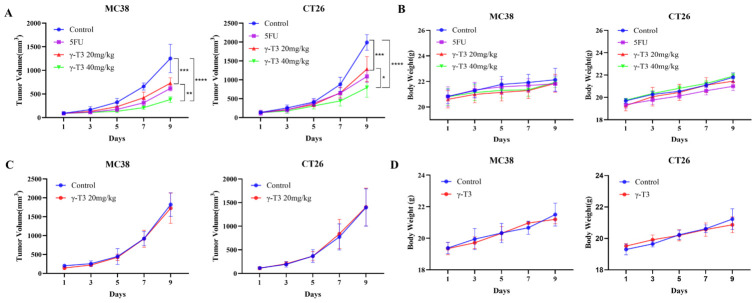
γ-T3 suppresses colorectal cancer growth more effectively in immunocompetent than in immunodeficient mice. (**A**) Tumour volume progression over time for each group in MC38 and CT26 syngeneic subcutaneous transplantation tumour models in immunocompetent mice. (**B**) Body weight change curves of each group in MC38 and CT26 models in immunocompetent mice. (**C**) Tumour growth curves of MC38 and CT26 subcutaneous tumours established in NOD-SCID immunodeficient mice and treated with vehicle or γ-T3 at 20 mg/kg. (**D**) Body weight change curves of each group in MC38 and CT26 models in NOD-SCID immunodeficient mice. *n* = 6, * *p* < 0.05, ** *p* < 0.01, *** *p* < 0.001, **** *p* < 0.0001.

To examine the in vivo tumour-suppressive effect of γ-T3 in colorectal cancer, we established MC38 and CT26 syngeneic subcutaneous tumour models in immunocompetent mice, with 5-FU used as a positive control. γ-T3 inhibited tumour growth in both models, with greater efficacy seen in the 40 mg/kg group (Figure 1A). No substantial loss in body weight was noted for any of the treatment cohorts, indicating that γ-T3 exhibited favourable preliminary tolerability (Figure 1B).

To determine whether the antitumour effect of γ-T3 depended on the host immune system, we further established MC38 and CT26 subcutaneous tumour models in NOD-SCID immunodeficient mice. Based on the results obtained in immunocompetent models, 20 mg/kg γ-T3, which had already shown antitumour activity, was selected as the validation dose. γ-T3 at 20 mg/kg failed to produce an antitumour effect in NOD-SCID mice comparable to that observed in immunocompetent hosts (Figure 1C), pointing to a strong connection between γ-T3’s tumour-suppressing effects in live subjects and the engagement of the host’s immune system. None of the groups showed significant weight reduction, further supporting its preliminary safety under the treatment conditions used in this study (Figure 1D).

### 3.2. γ-T3 Improves the Treatment Outcome of Anti-PD-1 Therapy in Colorectal Cancer Models

**Figure 2:**
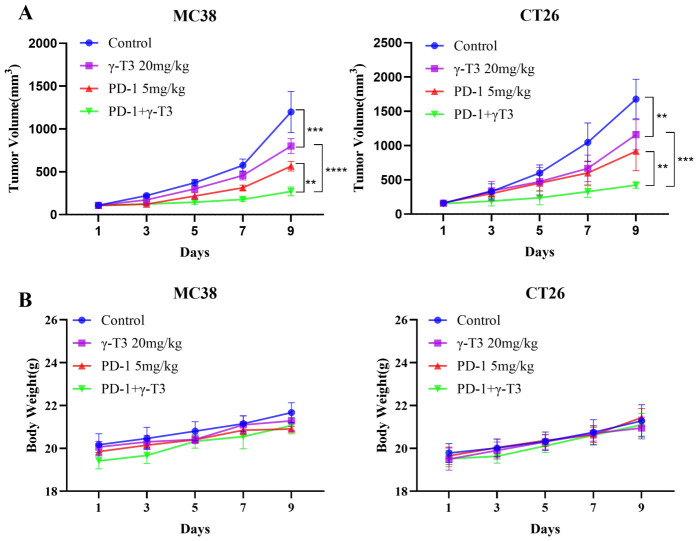
γ-T3 enhances the antitumour efficacy of PD-1 blockade in colorectal cancer models. (**A**) Tumour volume trajectories for various treatment arms within MC38 and CT26 subcutaneous tumour models. (**B**) Body weight changes of mice in different treatment groups in MC38 and CT26 subcutaneous tumour models. *n* = 6, ** *p* < 0.01, *** *p* < 0.001, **** *p* < 0.0001.

Given that the in vivo antitumour activity of γ-T3 was closely associated with the host immune system, we further evaluated whether γ-T3 may potentiate the treatment outcome of PD-1 pathway inhibition. In the MC38 and CT26 subcutaneous tumour models, γ-T3 monotherapy and anti-PD-1 monotherapy delayed tumour growth to varying degrees, whereas the combination treatment achieved stronger tumour control, as reflected by further suppression of tumour growth and reduced endpoint tumour volume (Figure 2A). None of the treated groups showed significant weight reduction, indicating that the combined therapy had favourable preliminary in vivo tolerability (Figure 2B). These results indicate that γ-T3 not only exerts immune-dependent antitumour activity but also enhances the antitumour efficacy of PD-1 blockade in colorectal cancer.

### 3.3. γ-T3 Remodels the Tumour Immune Microenvironment and Strengthens Antitumour Immune Responses

To elucidate the immunological basis underlying the γ-T3-mediated enhancement of PD-1 blockade efficacy, flow cytometric analysis was performed on subcutaneous MC38 and CT26 tumour tissues, as well as tumour-draining lymph nodes. The results showed that combined γ-T3 and PD-1 blockade did not significantly increase the overall proportions of CD8^+^ and CD4^+^ T cells within CD45^+^ immune cells in the tumour tissues (Figure S2A,B), suggesting that the immunopotentiating effect of γ-T3 is not primarily dependent on increased T cell infiltration.

**Figure 3:**
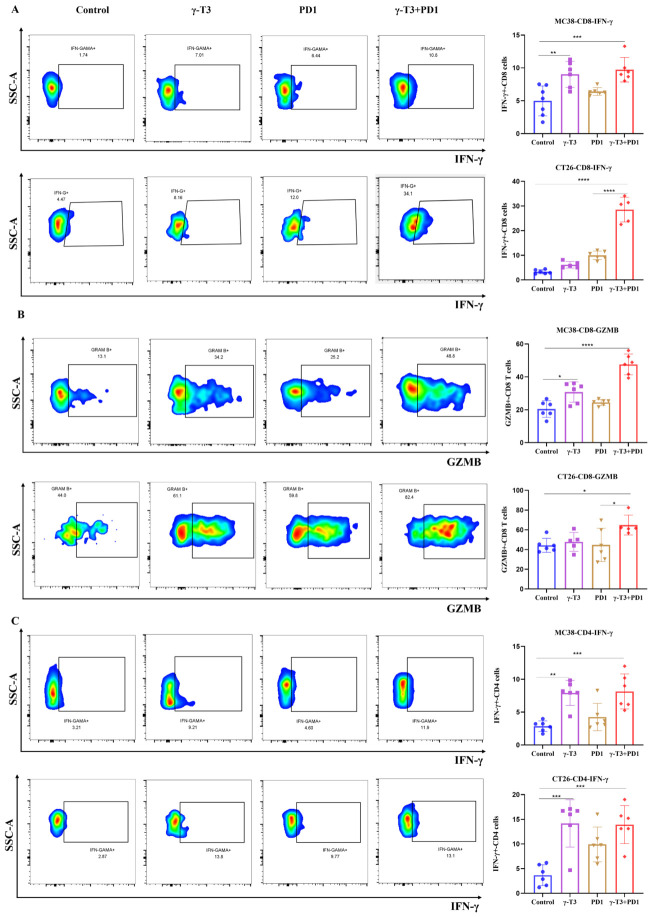
γ-T3 combined with PD-1 blockade enhances the effector function of tumour-infiltrating T cells. Flow cytometric assessment of cytokine-producing T cell subsets in subcutaneous MC38 and CT26 tumour tissues. (**A**) IFN-γ^+^CD8^+^ T cells—representative plots and statistical quantification. (**B**) GZMB^+^CD8^+^ T cells—representative plots and statistical quantification. (**C**) IFN-γ^+^CD4^+^ T cells—representative plots and statistical quantification. *n* = 6, * *p* < 0.05, ** *p* < 0.01, *** *p* < 0.001, **** *p* < 0.0001.

Further examination of T cell responses indicated that γ-T3 monotherapy and the combination regimen both enhanced the effector phenotype of tumour-infiltrating T cells. In the MC38 model, γ-T3 monotherapy and combination therapy increased the proportion of IFN-γ^+^CD8^+^ T cells to 1.81-fold and 1.95 times the value for the control cohort, respectively; in the CT26 model, these increases were 1.85-fold and 8.56-fold, respectively (Figure 3A). The combination therapy further augmented the proportion of GZMB^+^CD8^+^ T cells, increasing it to 2.33-fold and 1.47 times that of the control animals in the MC38 and CT26 models, respectively (Figure 3B). Additionally, both γ-T3 monotherapy and the combination therapy increased the proportion of IFN-γ^+^CD4^+^ T cells, reaching 2.77-fold and 2.85-fold in the MC38 model, and 3.86-fold and 3.79-fold in the CT26 model, respectively (Figure 3C). These findings indicate that γ-T3 improves the antitumour immune response primarily by enhancing the immune effector performance of T cells within tumours, rather than merely increasing T cell abundance.

Concurrently, both γ-T3 monotherapy and the combination regimen alleviated local immunosuppression within the tumour. γ-T3 monotherapy reduced the proportion of regulatory T cells (Tregs) by 31.29% and 44.25% in the MC38 and CT26 models, respectively, while the combination therapy achieved reductions of 57.79% and 64.19%, respectively (Figure S2C,D). The combination therapy also promoted a shift of tumour-associated macrophages (TAMs) toward an M1-like phenotype, increasing the proportion of M1 macrophages and the M1/M2 ratio to 1.66-fold and 1.47 times the level of control mice within the MC38 model, and to 1.95-fold and 2.17-fold in the CT26 model, respectively (Figure S2E,F).

In contrast, the combination therapy had a relatively limited effect on DC maturation markers (CD80, CD86, and MHC-II) in tumour-draining lymph nodes, with no consistent trend toward enhancement being observed (Figure S2K–M).

In summary, the γ-T3-mediated enhancement of PD-1 blockade efficacy is primarily associated with enhanced effector T cell function within the tumour, reduced Treg-mediated immunosuppression, and the repolarisation of TAMs toward an antitumour phenotype, rather than with a substantial increase in overall T cell infiltration or DC maturation.

### 3.4. CD8^+^ T Cells Are Essential for the Antitumour Activity of γ-T3

**Figure 4:**
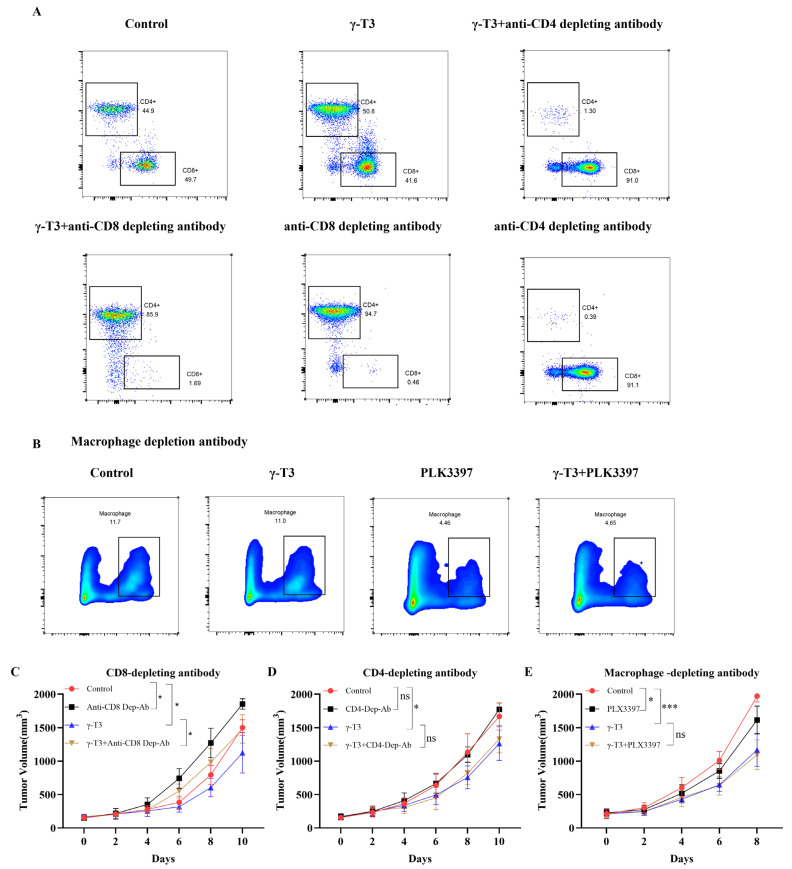
The in vivo antitumour activity of γ-T3 is primarily dependent on CD8^+^ T cells. (**A**) Typical flow cytometry images depicting the depletion efficiency of CD8^+^ T cells and CD4^+^ T cells. (**B**) Typical flow cytometry images depicting the depletion efficiency of macrophages. (**C**) Effect of γ-T3 on MC38 subcutaneous tumour growth under CD8^+^ T cell depletion. (**D**) Effect of γ-T3 on MC38 subcutaneous tumour growth under CD4^+^ T cell depletion. (**E**) Effect of γ-T3 on MC38 subcutaneous tumour growth under macrophage depletion. *n* = 6, ns indicates not significant, * *p* < 0.05, *** *p* < 0.001.

To further determine the immune cell dependency of the in vivo antitumour effect of γ-T3 and antibody- or drug-mediated cell depletion experiments were performed to deplete CD8^+^ T cells, CD4^+^ T cells, and macrophages. Flow cytometric validation demonstrated that the target cell populations were efficiently depleted following the respective treatments (Figure 4A,B), indicating that this experimental system is suitable for evaluating the necessity of distinct immune cell populations in the antitumour effect of γ-T3.

Under CD8^+^ T cell depletion, the γ-T3-mediated inhibition of tumour growth was markedly impaired, with tumour growth kinetics approaching those of the control group (Figure 4C), indicating that CD8^+^ T cells are essential core effector cells required for the in vivo antitumour effect of γ-T3. In contrast, following CD4^+^ T cell or macrophage depletion, γ-T3 retained a certain degree of tumour growth inhibition (Figure 4D,E), suggesting that although these cell types may participate in the modulation of the tumour immune microenvironment by γ-T3, they are not the primary limiting populations for its antitumour effect.

Collectively, these functional validation results further demonstrate that the in vivo antitumour effect of γ-T3 primarily depends on CD8^+^ T cell-mediated cytotoxic immune responses.

### 3.5. Single-Cell Transcriptomic Analysis Reveals γ-T3-Mediated Enhancement of Tumour Immune Visibility and T Cell Effector Remodelling

**Figure 5:**
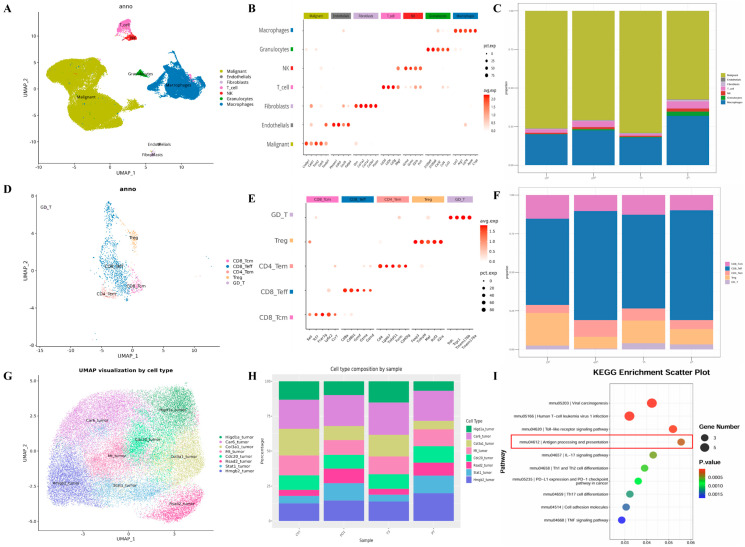
Single-cell transcriptomic analysis of the tumour immune microenvironment after γ-T3 treatment. (**A**) UMAP visualisation of major cell populations in MC38 subcutaneous tumour tissues, including malignant tumour cells and T cells. (**B**) Expression distribution of representative marker genes for major cell populations. (**C**) Relative proportions of major cell populations across different treatment groups. (**D**) UMAP visualisation of T cell subclusters, including CD8_Tcm, CD8_Teff, and CD4_Tem cells. (**E**) Expression distribution of representative marker genes for T cell subclusters. (**F**) Relative proportions of T cell subclusters across different treatment groups. (**G**) UMAP visualisation of malignant tumour cell subclusters. (**H**) Relative proportions of malignant tumour cell subclusters across different treatment groups. (**I**) KEGG-enrichment analysis of differentially expressed genes in malignant tumour cells associated with γ-T3 treatment. The antigen processing and presentation pathway is highlighted by the red box. T3 indicates γ-T3 treatment, PD1 indicates PD-1 blockade, and PT indicates combined γ-T3 and PD-1 blockade.

To further elucidate the potential mechanisms by which γ-T3 modulates the tumour immune microenvironment, we carried out single-cell RNA sequencing analysis on MC38 tumour tissues that had been engrafted subcutaneously. Following dimensionality reduction, clustering, and cell type annotation, the major cell populations identified within the tumour tissue included malignant cells, T cells, NK cells, granulocytes, macrophages, endothelial cells, and fibroblasts (Figure 5A). The expression profiles of conventional marker genes for every cell population were consistent with the annotated identities, supporting the reliability of cell classification (Figure 5B). Cellular composition analysis revealed that γ-T3 treatment did not markedly increase the overall proportion of T cells (Figure 5C), suggesting that its antitumour immune effects are not primarily dependent on promoting T cell infiltration.

Further subclustering of the T cell compartment identified distinct subsets, including central-memory-like CD8^+^ T cells (CD8_Tcm), effector-like CD8^+^ T cells (CD8_Teff), and effector-memory-like CD4^+^ T cells (CD4_Tem), Treg, and γδ T cells (Figure 5D,E). Proportional analysis showed that γ-T3 treatment increased the abundance of the CD8_Teff subset while reducing the proportion of Treg cells; combination with PD-1 blockade further augmented the CD8_Teff subset advantage (Figure 5F). These findings are consistent with the flow cytometric results, which demonstrated increased frequencies of IFN-γ^+^CD8^+^ and GZMB^+^CD8^+^ T cells, indicating that γ-T3 improves the functional composition of tumour-infiltrating T cells and enhances CD8^+^ T cell effector responses. SCENIC analysis further revealed that the regulon signatures associated with CD8_Teff cells following γ-T3 treatment and PD-1 blockade were not entirely congruent, suggesting that these interventions may influence CD8^+^ T cell function through distinct transcriptional regulatory programmes (Figure S3A,B).

At the tumour cell level, InferCNV analysis supported the reliability of malignant cell annotation (Figure S3C,D). Subsequent subclustering revealed differences in the composition of malignant cell subsets across treatment groups (Figure 5G,H). KEGG-enrichment analysis demonstrated that γ-T3 treatment-associated differentially expressed genes were significantly enriched in the antigen processing and presentation pathway, alongside multiple immune-related pathways (Figure 5I), indicating that γ-T3 treatment confers a more active immune recognition-associated transcriptional signature on tumour cells.

In summary, single-cell transcriptomic analysis indicates that γ-T3 correlates with a shift in the functional composition of tumour-infiltrating T cells and an enrichment of antigen processing and presentation programmes in tumour cells. These observations point to the conclusion that the antitumour immunomodulatory effects of γ-T3 may involve a combined interplay between enhanced tumour cell immune visibility and improved CD8^+^ T cell effector responses.

### 3.6. HSPA4 Is Identified as a Potential γ-T3-Binding Protein Associated with Antigen Presentation Regulation

**Figure 6:**
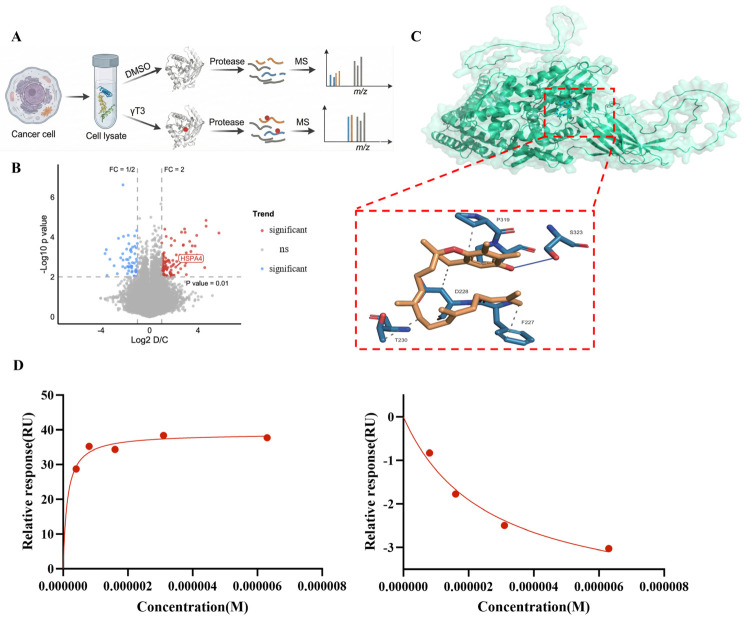
LiP-MS screening and validation identify HSPA4 as a potential γ-T3-binding protein. (**A**) Schematic workflow of the LiP-MS analysis. (**B**) Volcano plot showing the screening results of candidate proteins identified by LiP-MS. (**C**) Molecular docking model of γ-T3 with HSPA4. (**D**) SPR analysis of the binding of γ-T3 to wild-type and mutant HSPA4 proteins.

Based on the single-cell transcriptomic analysis, γ-T3 treatment was associated with MHC-I-related immune recognition features and alterations in transcriptional programmes involved in antigen processing. To further explore the potential basis of this effect at the level of protein structural response, we employed LiP-MS as a structural proteomics screening approach to identify candidate proteins that may undergo local conformational changes or altered protease accessibility following γ-T3 treatment (Figure 6A). LiP-MS analysis revealed significant changes in LiP peptide signals across multiple proteins after γ-T3 treatment, suggesting that γ-T3 may induce a broad protein structural response and potentially act through multiple targets or pathways. To avoid selective presentation of a single candidate protein, the integrated ranking of the major γ-T3-responsive candidate proteins is provided in Table S4.

Within this candidate protein list, HSPA4 was among the highly ranked γ-T3-responsive proteins (Figure 6B). Its selection for subsequent validation was not based on a single differential metric or molecular docking result, but rather on an integrated consideration of LiP-MS differential peptide signals, structural response features, protein–protein interaction network properties, and functional annotation. More importantly, HSPA4 belongs to the Hsp70/Hsp110 molecular chaperone network and is involved in protein folding, misfolded protein handling, protein quality control, and the maintenance of cellular proteostasis. As intracellular protein turnover, misfolded protein processing and proteasomal degradation may all influence the generation and presentation of MHC-I-associated antigenic peptides, HSPA4 is functionally relevant to the antigen-processing and MHC-I-related immune recognition phenotype observed in this study. Therefore, HSPA4 not only has high priority in LiP-MS-based integrated screening but also provides a biologically plausible candidate mechanistic entry point linking γ-T3-induced protein structural responses with enhanced MHC-I-related immune recognition.

Molecular docking analysis showed that γ-T3 could be accommodated within a local binding pocket of HSPA4, with a docking score of −5.513 kcal/mol. γ-T3 was predicted to form a hydrogen bond with Ser323 and to be positioned adjacent to residues, including Phe227, Asp228, Thr230, and Pro319, suggesting that Ser323 may contribute to stabilising the γ-T3–HSPA4 binding conformation (Figure 6C). Based on this prediction, we further used surface plasmon resonance (SPR) analysis to evaluate the binding responses of γ-T3 to wild-type HSPA4 and the S323A mutant.

SPR analysis showed that γ-T3 exhibited a concentration-dependent binding response to wild-type HSPA4 (HSPA4-WT). Steady-state fitting yielded an equilibrium dissociation constant (K_D) of 130.2 ± 34.6 nM, indicating detectable direct binding between γ-T3 and HSPA4 under the tested conditions (Figure 6D, left). In contrast, the HSPA4-S323A mutant did not show a positive concentration-dependent saturation profile comparable to that of HSPA4-WT. Its overall response signal was weaker, and no typical binding mode suitable for stable fitting was obtained; therefore, a reliable K_D value could not be determined (Figure 6D, right). These results indicate that the S323A mutation markedly weakens the measurable binding capacity of γ-T3 to HSPA4.

Taken together, the LiP-MS, molecular docking and SPR results suggest that HSPA4 may be a potential γ-T3-binding protein, and that Ser323 may be an important residue involved in maintaining the γ-T3–HSPA4 interaction.

### 3.7. γ-T3 Increases MHC-I Surface Expression and Sensitises Tumour Cells to Activated CD8^+^ T Cell-Mediated Growth Inhibition

To assess whether γ-T3 directly modulates the antigen-presenting capacity of tumour cells, we first evaluated its effects on the viability of MC38 and CT26 cells at 24 h and 72 h. γ-T3 treatment at 0, 15, 20, and 25 μM did not markedly reduce cell viability in either cell line at the examined time points. In contrast, higher concentrations, particularly 30–40 μM, decreased cell viability after prolonged exposure, especially at 72 h, indicating that high-dose γ-T3 may cause cytotoxicity or growth inhibition. Therefore, γ-T3 concentrations within 0–25 μM were selected as a non-overtly cytotoxic treatment range for subsequent experiments in order to minimise potential interference from cell death or growth inhibition in the analysis of MHC-I surface expression and antigen presentation.

**Figure 7:**
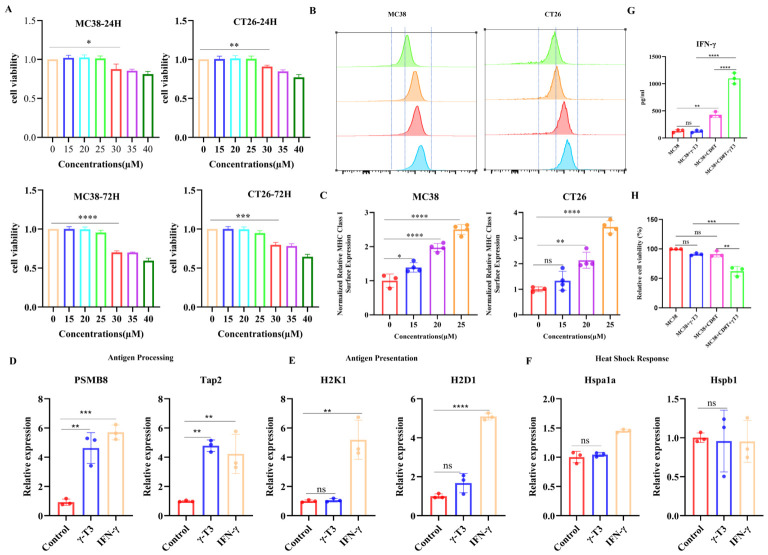
γ-T3 enhances MHC-I surface presentation on tumour cells and increases their susceptibility to CD8^+^ T cell-associated growth inhibition. (**A**) Relative survival of MC38 and CT26 cells following exposure to varying doses of γ-T3, used to evaluate the cytotoxicity of the concentration range applied in subsequent experiments. (**B**,**C**) Representative histograms and quantification of MHC-I abundance on MC38 and CT26 cells following exposure to γ-T3 treatment, as determined by flow cytometry. (**D**,**E**) qPCR analysis of the expression changes of the MHC-I heavy-chain genes H2-K1 and H2-D1, as well as the antigen processing- and peptide transport-related genes Psmb8 and Tap2, in MC38 and CT26 cells after γ-T3 treatment. (**F**) qPCR analysis of the effects of γ-T3 on the expression of typical heat-shock response-related genes, Hspa1a and Hspb1. (**G**) Measurement of IFN-γ levels in the culture supernatant after co-culture of MC38 cells with CD8^+^ T cells. (**H**) Relative viability of tumour cells after co-culture of MC38 cells with CD8^+^ T cells. *n* = 3, ns indicates not significant, * *p* < 0.05, ** *p* < 0.01, *** *p* < 0.001, **** *p* < 0.0001.

Flow cytometry revealed that γ-T3 treatment significantly upregulated MHC-I surface density on the membrane of MC38 and CT26 cells (Figure 7B,C), suggesting that γ-T3 enhanced the antigen-presenting phenotype of tumour cells. Further qPCR analysis showed that γ-T3 did not strongly promote the expression of the MHC-I heavy-chain genes H2-K1 and H2-D1, but it more prominently promoted the expression of the antigen processing- and peptide transport-related molecules Psmb8 and Tap2 (Figure 7D,E). In addition, γ-T3 did not broadly upregulate typical heat-shock response-related genes, such as Hspa1a and Hspb1 (Figure 7F), indicating that its regulation of the antigen presentation pathway was not simply attributable to non-specific heat-shock stress.

To additionally confirm the biological importance of this phenotype, we established a co-culture system of MC38 cells and CD8^+^ T cells. γ-T3 treatment alone did not induce obvious background IFN-γ secretion; however, in the presence of CD8^+^ T cells, γ-T3 treatment significantly increased IFN-γ levels in the co-culture system (Figure 7G) and further reduced MC38 cell viability (Figure 7H). Our data imply that γ-T3 enhances the susceptibility of tumour cells to recognition and growth inhibition by CD8^+^ T cells.

**Figure 8:**
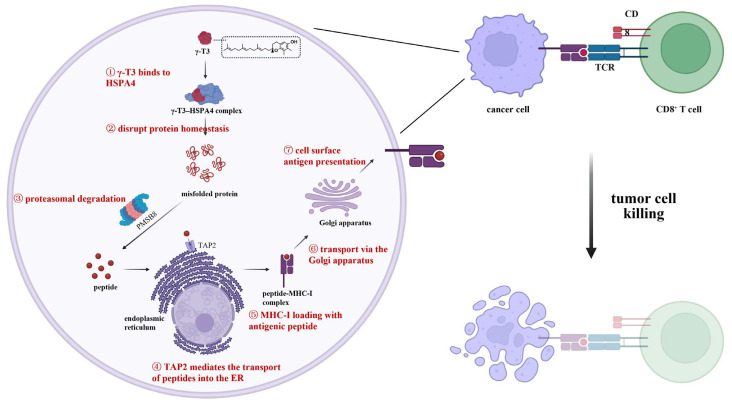
Proposed model by which γ-T3 enhances MHC-I-associated tumour immune visibility and CD8^+^ T cell-related antitumour immunity.

However, because defined peptide–MHC-I complexes and the MHC-I immunopeptidome were not directly analysed, these results should not be interpreted as direct evidence of enhanced antigen-specific presentation (Figure 8).

## 4. Discussion

This study systematically evaluated whether γ-T3 can act as a naturally derived immune-sensitising molecule to enhance the effectiveness of PD-1 pathway blockade against colorectal cancer. The results show that the in vivo antitumour activity of γ-T3 is clearly immune-dependent and that γ-T3 can augment the therapeutic benefit of PD-1 inhibition. Unlike prior investigations that largely centred on the direct effects of γ-T3 in inhibiting tumour-cell proliferation, inducing cell death, or modulating inflammation-related signalling pathways, the present study further suggests that the antitumour activity of γ-T3 may, at least in part, arise from its regulation of tumour immune recognition [33]. It should be noted that previous studies have implicated γ-T3 in multiple molecular processes, including p38/MAPK signalling, Bcl-2-associated survival or apoptotic regulation, and other cancer-related protein targets; therefore, the immune-recognition mechanism proposed here should be viewed as complementary to, rather than replacing, these previously described mechanisms. Specifically, γ-T3 enhanced CD8^+^ T cell-dependent antitumour immunity and increased MHC-I surface expression in tumour cells, indicating that γ-T3 may function not only as a direct antitumour bioactive molecule but also as an immunotherapeutic sensitiser by improving MHC-I-associated tumour immune visibility.

The core of tumour cell immune visibility lies in the formation and surface presentation of antigen peptide–MHC-I complexes. Previous studies have shown that B2M deficiency, defects in antigen processing and presentation, and impaired IFN-γ/JAK signalling can all compromise MHC-I-related antigen presentation, thereby promoting tumour immune evasion or resistance to immune checkpoint blockade [30,31,32]. In this study, γ-T3 upregulates Psmb8 and Tap2 and increases the membrane expression of MHC-I on tumour cells, indicating that it could potentiate the capacity of cancer cells to output pMHC-I signals to CD8^+^ T cells by improving antigen processing and peptide transport. Therefore, γ-T3 and PD-1 blockade may act on distinct stages of the tumour-immunity loop: γ-T3 may enhance the immune recognisability of tumour cells, whereas PD-1 blockade relieves inhibitory signalling on T cell effector function. This functional complementarity may provide a biological explanation for the improved antitumour response observed with the combination treatment. However, because formal synergy analyses were not performed, we describe this effect as an improved combination response rather than a validated synergistic interaction.

This mechanism also helps to explain how γ-T3 shapes the immune environment within tumours. The abundance of T cell accumulation does not automatically translate into effective antitumour immunity; rather, T cell functional status, the strength of antigen recognition, and the degree of local immunosuppression collectively determine the response to immunotherapy [34]. In the present work, combining γ-T3 with anti-PD-1 therapy did not primarily manifest as an overall increase in CD8^+^ or CD4^+^ T cell infiltration, but rather more prominently enhanced effector T cell function, reduced the proportion of Tregs, and promoted a shift of tumour-associated macrophages (TAMs) towards a state more favourable for antitumour responses. This suggests that γ-T3 does not act simply by expanding the scale of immune cell infiltration, but rather by improving the functional coupling between tumour immune visibility and T cell effector function, thereby enhancing the quality of the antitumour immune response. In other words, the immunomodulatory effect of γ-T3 is more closely related to improving the efficiency of immune recognition and optimising the local immune status, rather than broadly inducing immune cell entry into the tumour tissue.

Mechanistically, the γ-T3-induced enhancement of cell-surface MHC-I presentation may be associated with HSPA4-related proteostasis regulation. In this study, γ-T3 exerted only a limited effect on the transcription of H2-K1 and H2-D1, whereas it more markedly upregulated Psmb8 and Tap2, suggesting that its primary action may not be the direct transcriptional induction of MHC-I heavy chains, but rather the regulation of antigen processing and peptide transport. Previous studies have shown that MHC-I-presented peptides can be derived from misfolded proteins, short-lived proteins, and other products of protein quality-control processes [20,35]. In the present study, LiP-MS, molecular docking, and SPR analyses identified HSPA4 as a candidate binding protein of γ-T3, with Ser323 potentially involved in this interaction. Considering that γ-T3 did not markedly induce canonical heat-shock response genes, but enhanced Psmb8/Tap2 expression and cell-surface MHC-I presentation, we speculate that γ-T3 may influence HSPA4-related proteostasis processes, thereby promoting the entry of conformationally unstable or misfolded proteins into the proteasomal processing pathway and increasing the pool of endogenous antigenic peptides available for MHC-I presentation. This mechanistic interpretation links the natural small-molecule properties of γ-T3, HSPA4-associated protein quality control, and enhanced tumour-cell immune visibility, providing a plausible molecular basis for its immune-sensitising activity.

It should be noted that the antitumour activity of γ-T3 is characterised by a clear multi-target profile. Previous studies have largely been conducted in systems in which inhibition of tumour cell proliferation or induction of cell death served as the principal endpoints, showing that γ-T3 can affect p38 MAPK, NF-κB, Bcl-2/Bcl-xL, and caspase-related apoptotic pathways, as well as regulate molecules associated with cell proliferation, invasion, and angiogenesis [36]. In contrast to these studies, which mainly emphasised direct cytotoxic effects, the present study employed a low-dose γ-T3 treatment condition that did not induce substantial cell death, and focused on its effects on tumour immune recognition, MHC-I antigen presentation, and CD8^+^ T cell-associated antitumour responses. Therefore, HSPA4-associated regulation of protein homeostasis may be more appropriately considered as one of the candidate mechanisms by which γ-T3 enhances tumour immune recognition under low-dose conditions. Together, with previously reported cytotoxicity-related mechanisms, this may contribute to the multi-layered mechanistic basis underlying the antitumour effects of γ-T3.

Of note, in the present work, HSPA4 is more appropriately understood as a candidate key molecule in the process of γ-T3-related regulation of antigen presentation, rather than being overinterpreted as a fully validated direct functional target. This interpretation also does not exclude the possibility that other γ-T3-responsive targets or pathways, including those reported previously, may contribute to the observed antitumour and immunomodulatory effects. On the one hand, results from LiP-MS, molecular docking, and SPR support a potential interaction between γ-T3 and HSPA4. On the other hand, the current evidence primarily points to binding association and mechanistic relevance, which are insufficient to prove that HSPA4 is the sole essential upstream factor required for γ-T3-enhanced MHC-I antigen presentation. Therefore, further validation through HSPA4 knockdown/knockout, wild-type HSPA4 rescue, and Ser323 mutant rescue experiments would help clarify the causal role of the γ-T3-HSPA4 interaction in the enhancement of antigen presentation.

This study still has several limitations. First, whether γ-T3 alters the composition, abundance, and immunogenicity of the MHC-I immunopeptidome requires more direct validation through immunopeptidomic analysis [16]. Second, the proposed mechanism is currently based mainly on murine colorectal cancer models and mouse tumour cell lines; its applicability to human colorectal cancer samples and different molecular subtypes remains to be further evaluated. In addition, the in vivo pharmacokinetic properties, bioavailability, long-term safety, and optimal therapeutic window for combining γ-T3 with PD-1 blockade therapy require systematic investigation in future studies [37].

Overall, this study suggests that γ-T3, as a naturally derived antitumour immunomodulatory molecule, may enhance MHC-I-associated tumour immune visibility and improve the sensitivity of tumour cells to activated CD8^+^ T cell-mediated immune pressure. HSPA4 was identified as a candidate γ-T3-associated protein that may be linked to these immune-recognition changes, but its causal contribution remains to be functionally validated. These findings broaden the antitumour pharmacological significance of γ-T3 and provide a potential intervention strategy for combining naturally derived bioactive molecules with immune checkpoint blockade in colorectal cancer treatment.

## 5. Conclusions

In conclusion, this study demonstrates that γ-T3, a naturally occurring vitamin E isoform, suppresses colorectal cancer growth in mice and enhances the antitumour activity of PD-1 pathway inhibition. Unlike previous studies that primarily focused on its direct antitumour, anti-inflammatory, or antioxidant activities, this study shows that γ-T3 may also serve as a natural-source antitumour immunomodulator that contributes to immunotherapeutic sensitisation by improving tumour immune recognition. Mechanistically, γ-T3 enhanced the expression of antigen processing- and peptide transport-related molecules, including Psmb8 and Tap2, and increased tumour-cell surface MHC-I expression. HSPA4 was identified as a candidate γ-T3-associated protein by LiP-MS, molecular docking, and SPR analyses, providing a potential mechanistic clue linking γ-T3 to proteostasis-related regulation of antigen presentation. However, the causal role of HSPA4 in γ-T3-induced enhancement of antigen presentation remains to be established.

In parallel, γ-T3 enhances CD8^+^ T cell effector function, reduces Treg-associated immunosuppression, and promotes the polarisation of tumour-associated macrophages towards a phenotype more conducive to antitumour responses, thereby improving the local antitumour immune microenvironment. Overall, this study extends the pharmacological significance of γ-T3 from a direct anti-tumour natural compound to a potential immune-sensitising agent, and it supports the further development of natural-source bioactive molecules as adjuvant strategies for improving immune checkpoint blockade in colorectal cancer therapy.

## Data Availability

The original contributions presented in this study are included in the article/Supplementary Material. Further inquiries can be directed to the corresponding authors. During the preparation of this manuscript, ChatGPT (OpenAI, GPT-5.5 Thinking) was used solely to assist with grammatical refinement and language clarity. The authors reviewed and edited the manuscript and take full responsibility for the final content.

## Supplementary Materials

The following supporting information can be downloaded at: https://www.mdpi.com/article/10.3390/biom16070964/s1, Figure S1: Representative gating strategies for flow cytometry analysis; Figure S2: Flow-cytometric analysis of tumour immune-cell infiltration, tumour-associated macrophage polarisation and tumour-draining lymph-node dendritic-cell maturation; Figure S3: Transcriptional regulatory network analysis of CD8_Teff cells and validation of malignant tumour cell annotation; Table S1: Major Experimental Reagents; Table S2: Sample pooling strategy and quality-control metrics for exploratory single-cell RNA sequencing; Table S3: Primer sequences for qPCR amplification of target genes; Table S4: Integrated ranking of major γ-T3-responsive candidate proteins.

## Abbreviations

γ-T3	Gamma-tocotrienol
CRC	Colorectal cancer
PD-1	Programmed cell death protein 1
MHC-I	Major histocompatibility complex class I
HSPA4	Heat shock protein family A member 4
dMMR	Deficient mismatch repair
pMMR	Proficient mismatch repair
MSS	Microsatellite stable
MSI-H	Microsatellite instability-high
5-FU	5-Fluorouracil
