# Supplementary material for: γ-Tocotrienol Sensitises Colorectal Cancer to PD-1 Blockade by Enhancing MHC-I-Associated Tumour Immune Visibility and CD8+ T Cell-Related Antitumour Immunity
Source: Biomolecules. 2026 Jun 30;16(7):964. doi: 10.3390/biom16070964 (PMC13406994; doi:10.3390/biom16070964)
Supplement: Supplementary file 1 [file biomolecules-16-00964-s001.zip › Supplementary Data.pdf]

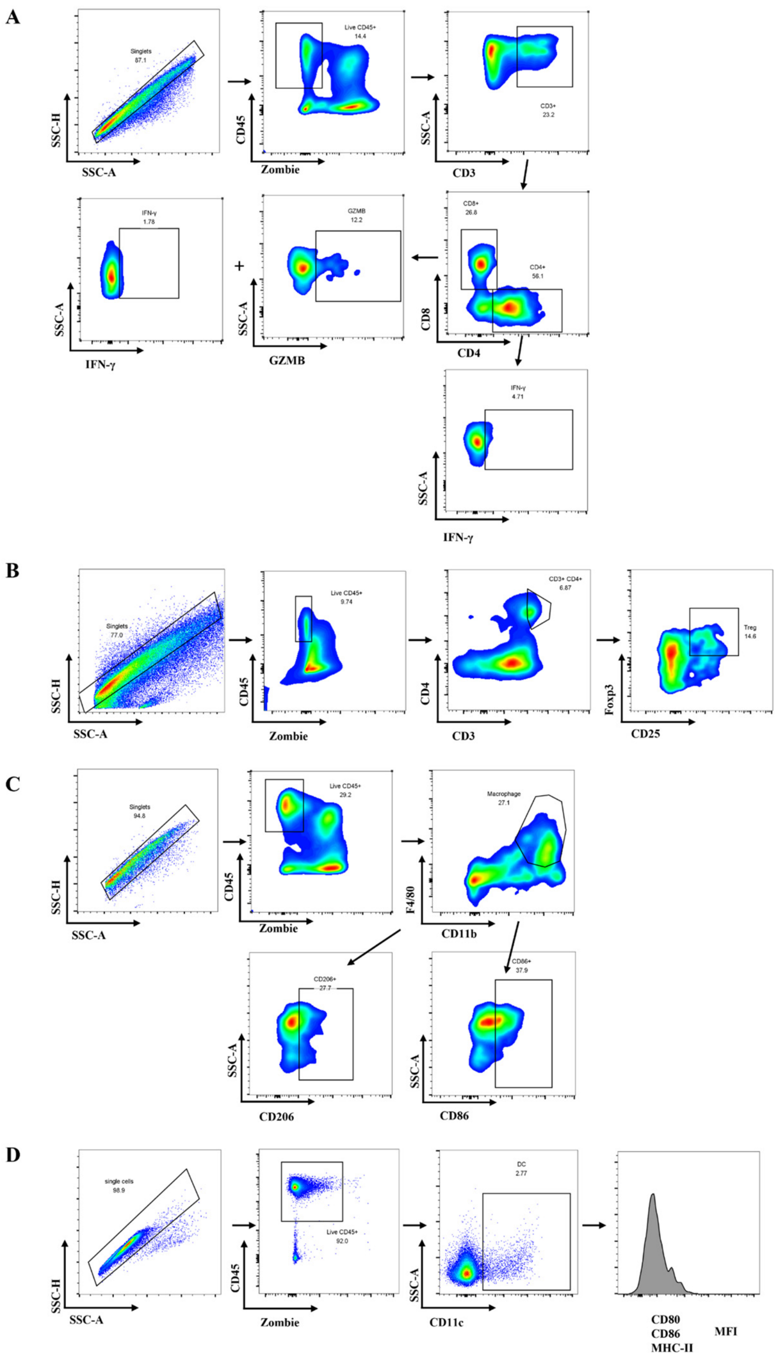


**Figure S1**. Representative gating strategies for flow cytometry analysis.

(**a**) Representative gating strategy for tumour-infiltrating CD4$^+$ and CD8$^+$ T cells, as well as IFN-$\gamma^+$ and GZMB$^+$ effector T cells. (**b**) Representative gating strategy for Treg cells in tumour tissues. (**c**) Representative gating strategy for tumour-associated macrophages and CD86$^+$ M1-like and CD206$^+$ M2-like TAMs. (**d**) Representative gating strategy for identifying dendritic cells in draining lymph nodes and analysing the expression of the maturation-associated markers CD80, CD86, and MHC-II.

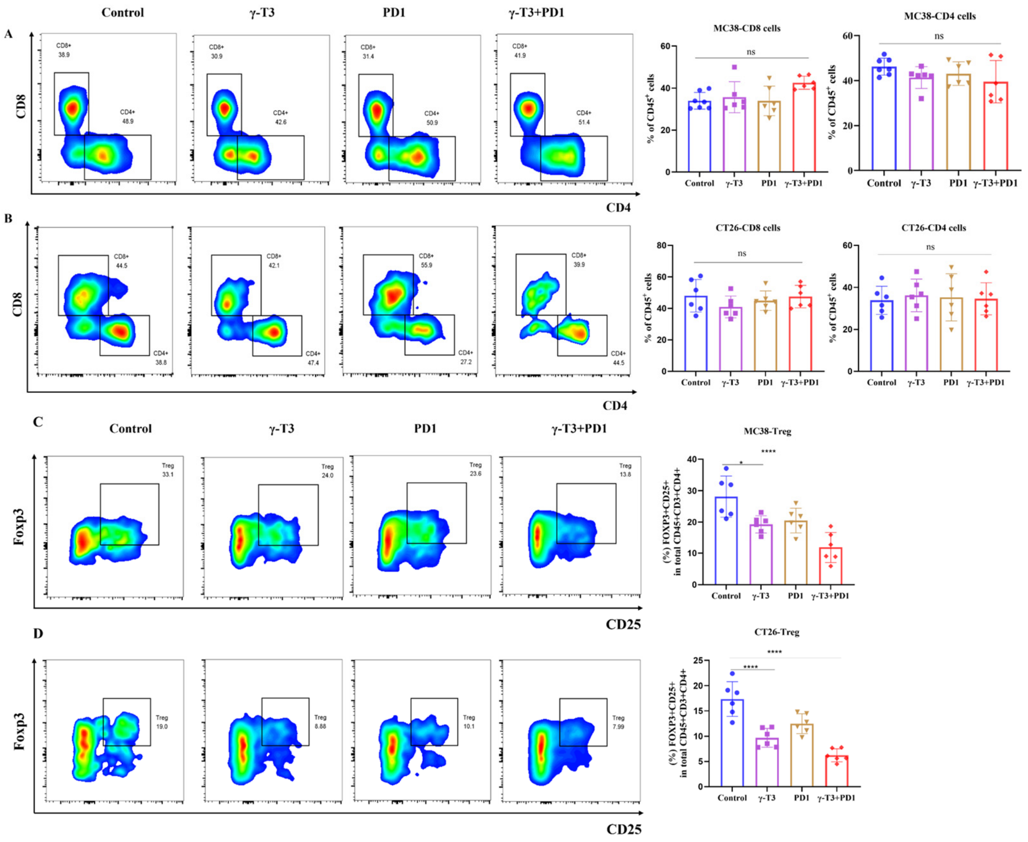

A
B
C
D
Control
γ-T3
PD1
γ-T3+PD1
CD8
CD4
Foxp3
CD25
MC38-CD8 cells
MC38-CD4 cells
CT26-CD8 cells
CT26-CD4 cells
MC38-Treg
CT26-Treg
% of CD45+ cells
(%) FOXP3+CD25+ in total CD45+CD3+CD4+
ns

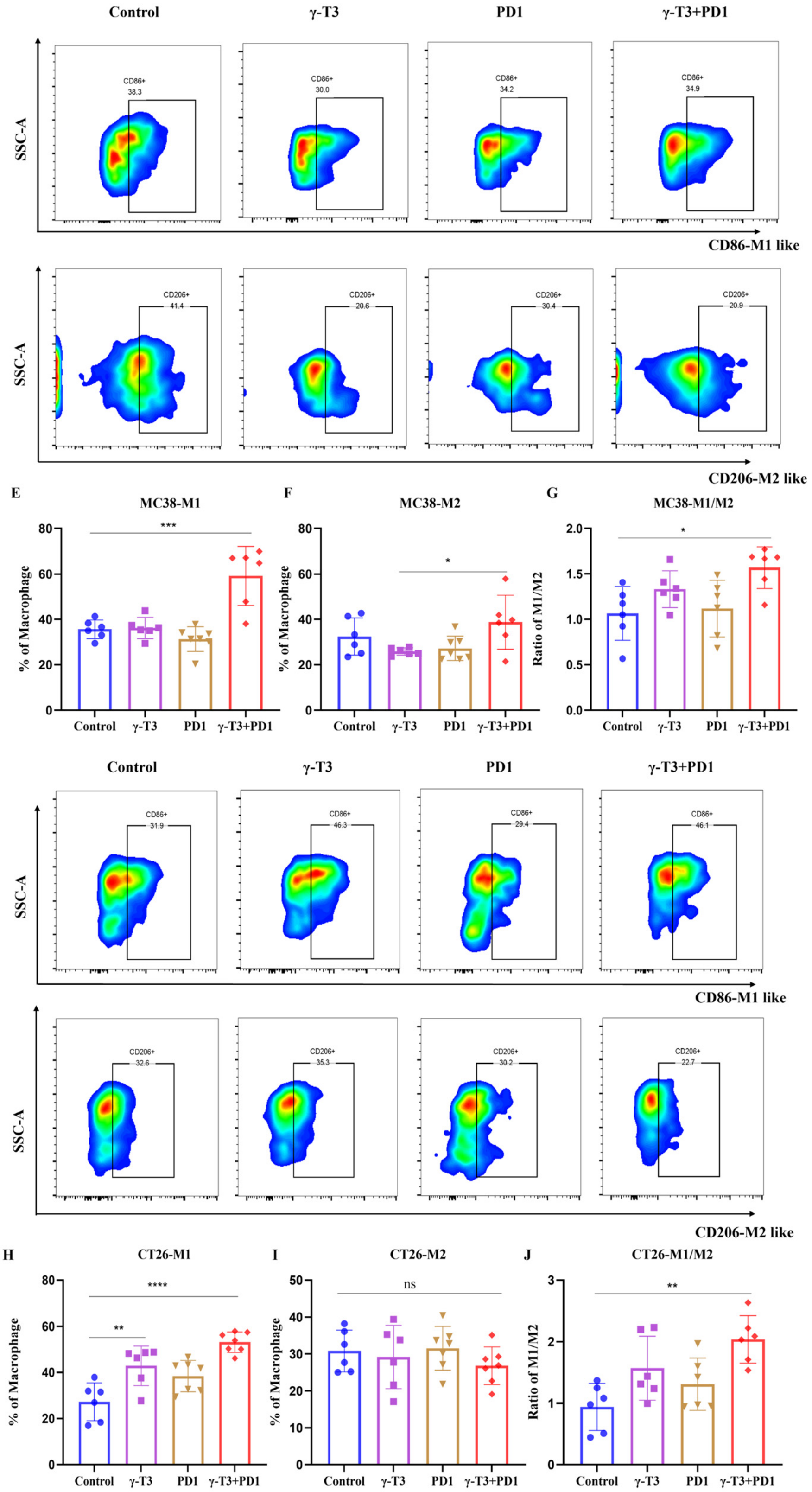
Control
γ-T3
PD1
γ-T3+PD1
SSC-A
CD86+
38.3
CD86+
30.0
CD86+
34.2
CD86+
34.9
CD86-M1 like
SSC-A
CD206+
41.4
CD206+
20.6
CD206+
30.4
CD206+
20.9
CD206-M2 like
E
MC38-M1
***
% of Macrophage
Control
γ-T3
PD1
γ-T3+PD1
F
MC38-M2
*
% of Macrophage
G
MC38-M1/M2
*
Ratio of M1/M2
Control
γ-T3
PD1
γ-T3+PD1
SSC-A
CD86+
31.9
CD86+
46.3
CD86+
29.4
CD86+
46.1
CD86-M1 like
SSC-A
CD206+
32.6
CD206+
35.3
CD206+
30.2
CD206+
22.7
CD206-M2 like
H
CT26-M1
****
**
% of Macrophage
I
CT26-M2
ns
% of Macrophage
J
CT26-M1/M2
**
Ratio of M1/M2

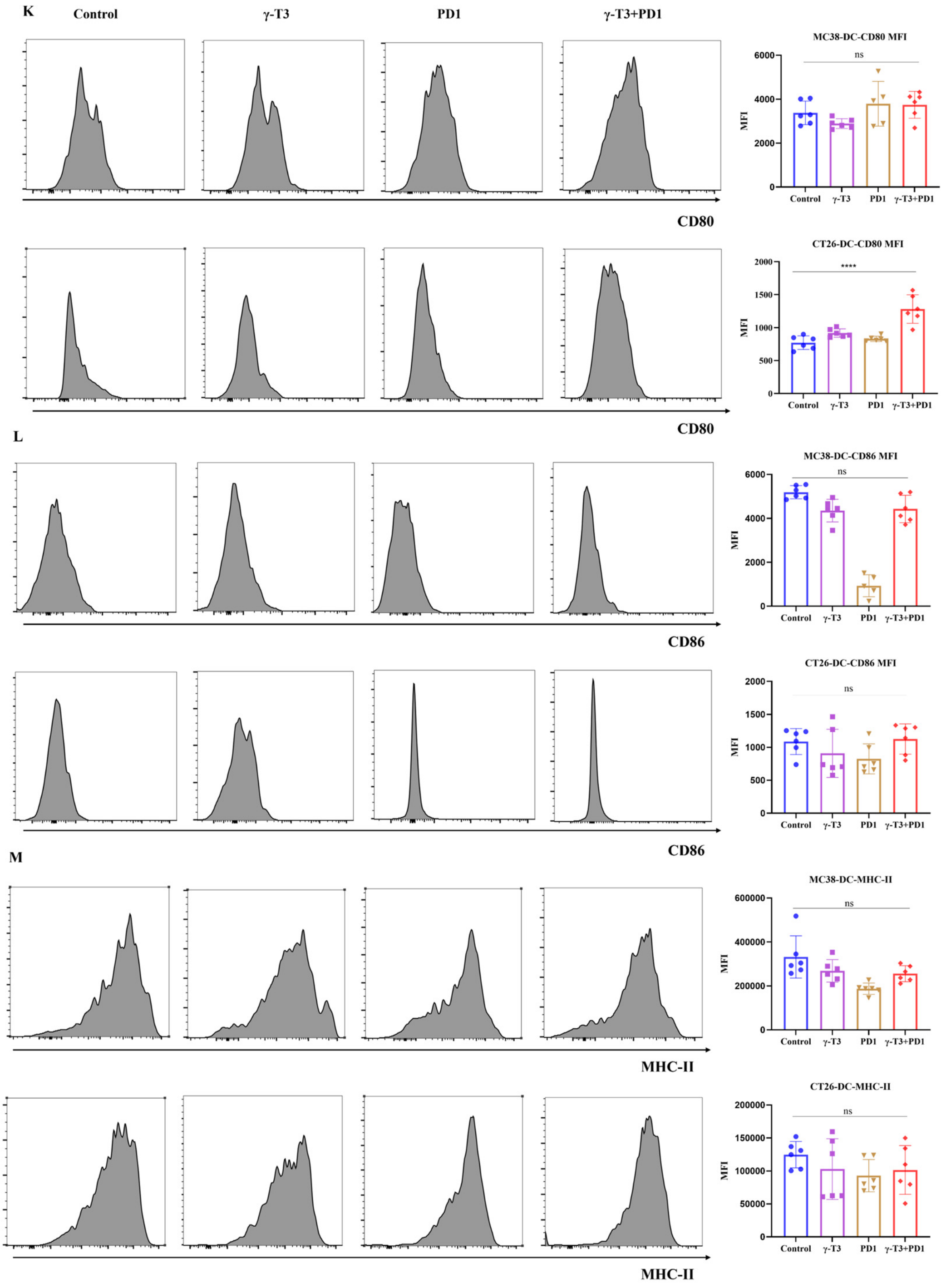


**Figure S2.** Flow-cytometric analysis of tumour immune-cell infiltration, tumour-associated macrophage polarisation and tumour-draining lymph-node dendritic-cell maturation. (**a, b**) Representative flow cytometry plots and quantitative analysis of the proportions of $CD4^{+}$ and $CD8^{+}$ T cells among $CD45^{+}$ tumour-infiltrating immune cells in MC38 (**a**) and CT26 (**b**) tumour tissues. (**c, d**) Representative flow cytometry plots and quantitative analysis of Treg cells in MC38 (**c**) and CT26 (**d**) tumour tissues. **e–g** Quantitative analysis of the proportion of $CD86^{+}$ M1-like tumour-associated macrophages (**e**), the proportion of $CD206^{+}$ M2-like

tumour-associated macrophages (**f**), and the M1-like/M2-like tumour-associated macrophage ratio (**g**) in MC38 tumour tissues. **h–j** Quantitative analysis of the proportion of $CD86^{+}$ M1-like tumour-associated macrophages (**h**), the proportion of $CD206^{+}$ M2-like tumour-associated macrophages (**i**), and the M1-like/M2-like tumour-associated macrophage ratio (**j**) in CT26 tumour tissues. K–M Quantitative analysis of the mean fluorescence intensity of CD80 (**k**), CD86 (**l**), and MHC-II (**m**) in dendritic cells from the tumour-draining lymph nodes of MC38 and CT26 tumour-bearing mice. *n = 6, ns indicates not significant,* $^{*}p < 0.05$, $^{**}p < 0.01$, $^{***}p < 0.001$, $^{****}p < 0.0001$

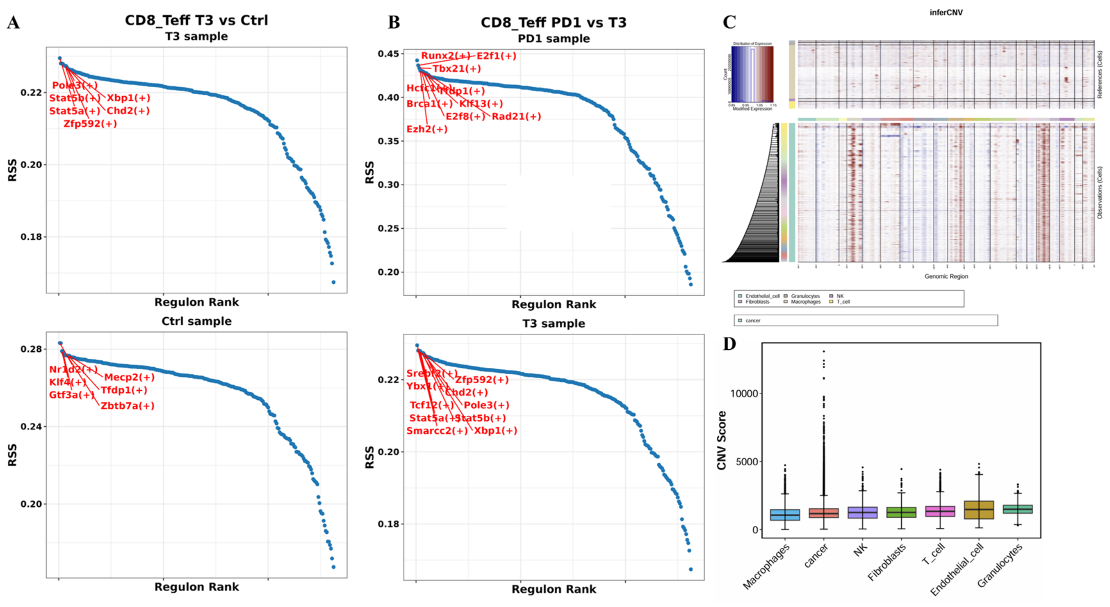


**Figure S3.** Transcriptional regulatory network analysis of CD8_Teff cells and validation of malignant tumour cell annotation. (**a**) SCENIC regulon ranking analysis of CD8_Teff cells from the γ-T3 and control groups, showing representative regulons with high activity in each group. (**b**) SCENIC regulon ranking analysis of CD8_Teff cells from the PD-1 blockade and γ-T3 groups, showing distinct treatment-associated regulatory features. (**c**) Infer CNV analysis showing that malignant tumour cells exhibited marked copy number variation patterns compared with the reference cell populations. (**d**) Distribution of CNV scores across different cell populations, supporting the reliability of malignant tumour cell annotation.

**Table S1** Major Experimental Reagents

| Reagents | **manufacturer** |
|---|---|
| Zombie BV510 Live/Dead dye | BioLegend |
| CD45 APC antibody | BioLegend |
| CD45 FITC antibody | BD Biosciences |

| Reagents | **manufacturer** |
|---|---|
| CD3 FITC antibody | BioLegend |
| CD3 BV421 antibody | BioLegend |
| CD4 PE antibody | BioLegend |
| CD8 PE-Cy5.5 antibody | BioLegend |
| CD25 BV421 antibody | BioLegend |

**Table S3** Primer sequences for qPCR amplification of target genes

| **primer name** | **primer sequence（5′→3′）** |
|---|---|
| M-β-actin-S | GTGACGTTGACATCCGTAAAGA |
| M-β-actin-A | GTAACAGTCCGCCTAGAAGCAC |
| M-Tap2-S | TATGGGCCTGAGGGACTGTGA |
| M-Tap2-A | TCCAGTTCTGTAGGGCCTGT |
| M-H2K1-S | CGTTGCTGTTCTGGTTGTCCT |
| M-H2K1-A | GGGTCATGAACCATCACTTTACAAT |
| M-H2D1-S | CGTTGCTGTTCTGGGTGTCCT |
| M-H2D1-A | CTGAACCCAAGCTCACAGGG |
| M-Psmb8-S | ATTCCTGAGGTCCTTTGGTGG |
| M-Psmb8-A | CCATTCCGAAGATAATACAACCTG |
| M-Hspa1a-S | GGTTCGAAGAGCTGTGCTCA |
| M-Hspa1a-A | ATCTGCGCCTTGTCCATCTT |
| M-Hspb1-S | CGGAGATCACCATTCCGGTT |
| M-Hspb1-A | CCAGCAATGGCTATGGGAGA |
